# Effect of Specific Retinoic Acid Receptor Agonists on Noise-Induced Hearing Loss

**DOI:** 10.3390/ijerph16183428

**Published:** 2019-09-16

**Authors:** Sang Hyun Kwak, Gi-Sung Nam, Seong Hoon Bae, Jinsei Jung

**Affiliations:** 1 Department of Otorhinolaryngology, Yonsei University College of Medicine, Seoul 03722, Korea; ENDLESSRAIN01@yuhs.ac (S.H.K.); BSHSAP1@yuhs.ac (S.H.B.); 2 Department of Otorhinolaryngology, Chonbuk National University College of Medicine, Jeonju 54907, Korea; jjs1980@daum.net

**Keywords:** Noise-induced hearing loss, sensorineural hearing loss, retinoic acid, selective RAR agonists

## Abstract

Noise is one of the most common causes of hearing loss in industrial countries. There are many studies about chemical agents to prevent noise-induced hearing loss (NIHL). However, there is no commercially available drug yet. Retinoic acid is an active metabolite of Vitamin A; it has an anti-apoptic role in NIHL. This study aims to verify the differences among selective agonists of retinoic acid receptors (RARs) in NIHL. All-trans retinoic acid (ATRA), AM80 (selective retinoic acid receptor α agonist), AC261066 (Selective retinoic acid receptor β1 agonist), and CD1530 (Selective retinoic acid λ agonist) were injected to 6–7 weeks old CJ5BL/6 mice before noise (110 dB for 3 h) exposure. In the auditory brainstem response test pre-, post 1, 3, and 7 days after noise exposure, not only ATRA but all kinds of selective RAR agonists showed protective effects in hearing threshold and wave I amplitude. Though there was no significant difference in the level of protective effects between agonists, α agonist showed the most prominent effect in preserving hearing function as well as outer hair cells after noise exposure. In conclusion, selective agonists of RAR demonstrate comparable protective effects against NIHL to retinoic acid. Given that these selective RAR agonists have less side effects than retinoic acid, they may be promising potential drugs against NIHL.

## 1. Introduction

Noise-induced hearing loss (NIHL) is a common form of hearing loss inin industrial countries. In particular, enhanced exposure to noise in work places can result in hearing loss. The number of patients with NIHL has greatly increased, making it a significant public health problem. The World Health Organization estimates that 1.1 billion young people worldwide could be at risk of hearing loss [1].

The mechanisms of NIHL comprise both direct mechanical damage and metabolic damage. Excessive noise can cause damage to middle ear structures, round and oval window membranes, and the organ of Corti. The outer hair cells of the cochlea are highly vulnerable to noise exposure. Noise can distort the stereocilia of hair cells, causing defects in mechanotransduction. Free radicals (e.g., reactive oxygen species) can damage the cell membrane through necrosis or apoptosis of neighboring cells [2].

Several compounds have been developed to protect against NIHL; however, there is no commercially available drug yet. Retinoic acid (RA), an active metabolite of vitamin A, enables hearing recovery in mouse models of NIHL. RA can inhibit the JNK pathway of apoptosis [3], which is activated in stressed inner ear cells [4]. Another study has shown that RA-induced peroxiredoxin 6 expression can contribute to recovery from noise-induced temporary hearing threshold shift [5]. RA plays various roles in cell induction, differentiation, and development [6]; RA binds to RA receptors (RAR) and retinoid x receptors (RXR), both of which serve as potential therapeutic targets in the inner ear. RAR α and λ transcripts are found in the organ of Corti and the spiral ganglion whereas RAR β1 transcripts are found in mesenchymal-derived tissue in the inner ear of mice [7]. Notably, RAR α and λ are required for inner ear development and have an essential role in the initial differentiation in mice [8].

In this study, we examined the effect of selective administration of RAR agonists to prevent NIHL.

## 2. Materials and Methods

### 2.1. Animals

C57BL/6 mice (6–7 weeks old; Orient Bio, GyeongGi province, Korea) were housed and maintained according to the Yonsei University Health System animal research requirements; all procedures were approved by the Yonsei University Health System Institutional Animal Care and Use Committee (IACUC Approval Number 2017-0181). Mice were fed ad libitum and housed in cages in an environmentally controlled room under a 12-h light cycle. A total of 40 mice were used, 8 mice per group, randomly distributed.

### 2.2. Noise Generation

White noise (300–10,000 Hz) was generated by a personal computer and an amplifier (R-399, Inter M, Seoul, Korea) and delivered through speakers (290-8L, Altec Lansing, Oklahoma City, OK, USA) in a noise booth. Mice were continuously exposed to 110 dB peak equivalent SPL for one 3-h session. It was considered that noise exposure of 110 dB SPL for 3 h is reasonable to induce NIHL based on a review of the literature [5,9].

### 2.3. All-Trans Retinoic Acid (ATRA) and Selective RAR Agonist Treatment

ATRA, AM80 (RAR α agonist), AC261066 (RAR β1 agonist), CD1530 (RAR λ agonist), and dimethylsulfoxide (DMSO, control) were used to prevent cochlear damage during noise exposure. They were all dissolved in DMSO at final concentrations of 20 mmol. ATRA and each selective agonist were administered to mice 1 day before noise exposure and 2 h before noise exposure via intraperitoneal (IP) injection. The control groups underwent IP injection of DMSO or water ( 60 µL). 

### 2.4. Audiologic Evaluation

The hearing level of each mouse was checked by measuring the auditory brainstem response (ABR) threshold with a TDT auditory evoked potential workstation (Tucker-Davis Technologies, Alachua, FL, USA) as described previously [10]. Mice were anesthetized by IP injection of ketamine hydrochloride (30 mg/kg) and xylazine (2 mg/kg). Both ears of each mouse were stimulated with an ear probe sealed in the ear canal. The ABRs to click and tone stimuli were recorded, and thresholds were obtained for each ear. ABRs were measured before noise exposure, immediately after noise exposure, 2 days after noise exposure, and 6 days after noise exposure.

### 2.5. Cochlear Cell Survival

One week after noise exposure, mice were euthanized with CO_2_ gas and the cochlea was quickly removed from each mouse. A whole mount preparation of the cochlea was performed. All cochlear tissues were separated into apical, middle, and basal turns. After dissection, middle turn was immunostained with fluorescently tagged antibodies. The rabbit myosin 7a antibody was used as primary antibody to stained cochlear hair cells, and 4′,6-diamidino-2-phenylindole (DAPI) was used to counterstain nuclei. All cochlear turns were examined under a Zeiss LSM 700 confocal microscope [10,11]. Outer hair cells were counted and compared between groups.

### 2.6. Statistics

Statistical analyses of ABR threshold shift between groups were performed with the Kruskal-Wallis and Mann-Whitney tests for comparisons between pairs of groups, using IBM SPSS Statistics for Windows, Version 22.0 (Armonk, NY, USA) and GraphPad Prism (Version 5) (La Jolla, CA, USA). Differences between mean values were considered statistically significant at *p* < 0.05.

## 3. Results

**Figure 1:**
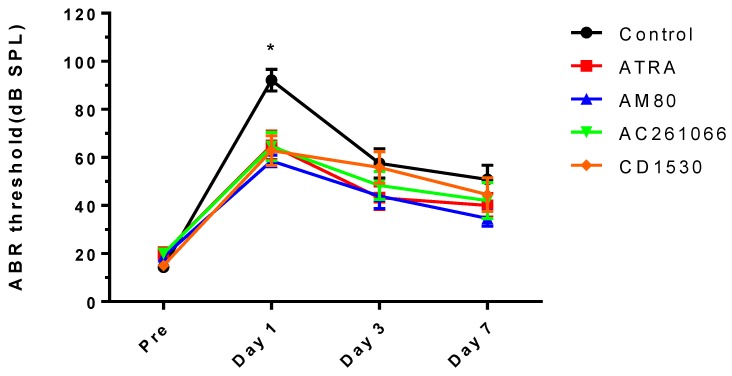
Changes in click-evoked auditory brainstem response (ABR) threshold. Pre indicates before noise exposure, Day 1 is immediately after noise exposure. There was a significant difference among the groups immediately after noise exposure. * *p* < 0.05.

All five groups demonstrated healthy hearing thresholds before noise exposure. After noise exposure, the control group (DMSO injection via IP) showed 92.1 ± 16.8 dB of hearing loss in click-evoked ABR, with an elevated hearing threshold immediately after noise exposure. The ABR thresholds of ATRA-treated and RAR agonist-treated mice were lower than those of the control group; there was a statistically significant difference in click-evoked ABR (Figure 1).

**Figure 2:**
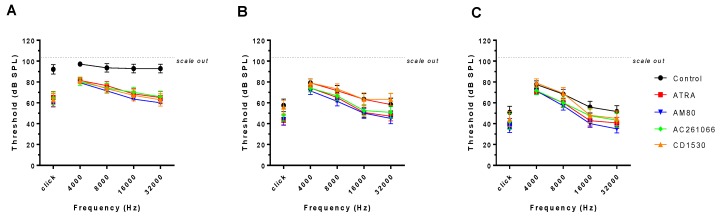
Click-evoked auditory brainstem response (ABR) thresholds immediately after noise exposure at Day 1 (**A**), 3 (**B**) and 7 (**C**). The hearing thresholds were significantly lower in groups treated with all-trans retinoic acid (ATRA) and all selective retinoic acid receptor (RAR) agonists than in the control group at every frequency, especially high frequency at Day 1(**A**). (*p* < 0.05) ABR thresholds began to recover in all groups at Day 3 and Day 7.

Figure 2 shows the ABR thresholds of all groups at day 1, 3, and 7. The ATRA-injected group showed 65.0 ± 22.1 dB of hearing loss; the AM80-injected group showed 58.6 ± 9.1 dB of hearing loss; the AC261066-injected group showed 64.6 ± 20.4 dB of hearing loss; and the CD1530-injected group showed 62.9 ± 23.1 dB of hearing loss. In tone-evoked ABR especially, hearing threshold was lower at low frequencies, 4000 and 8000 Hz, which indicates that the protective effects of RAR agonists mostly affect high frequencies. At day three, threshold shift in ABR showed recovery in five groups; this recovery differed among control, ATRA-treated, and RAR agonist-treated groups, but the statistical evidence for this difference was weak. At day seven, the control group showed partial recovery of ABR threshold, 50.8 ± 20.5 dB of hearing loss. All RAR agonist-treated groups showed recovery from noise exposure.

**Figure 3:**
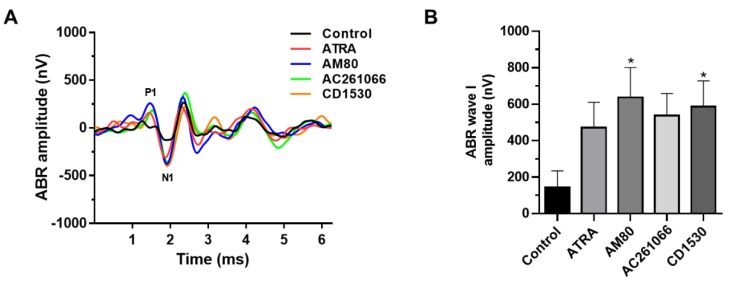
Comparison of wave I amplitude in auditory brainstem response (ABR). In click-evoked ABR (80 dB HL stimulus), wave I amplitude from P1 to N1 was acquired (**A**). The amplitude of wave I was significantly higher in the groups treated with ATRA and RAR agonists than the control group one day after noise exposure (**B**). Statistical analysis was performed with the reference value of lane 1. * *p* < 0.05.

We also examined the amplitude of wave I in ABR. As seen in Figure 3, the amplitude of wave I was significantly higher in the treatment groups with RAR agonists (AM80 and CD1530) compared to the control group after noise exposure (Figure 3A,B).

**Figure 4:**
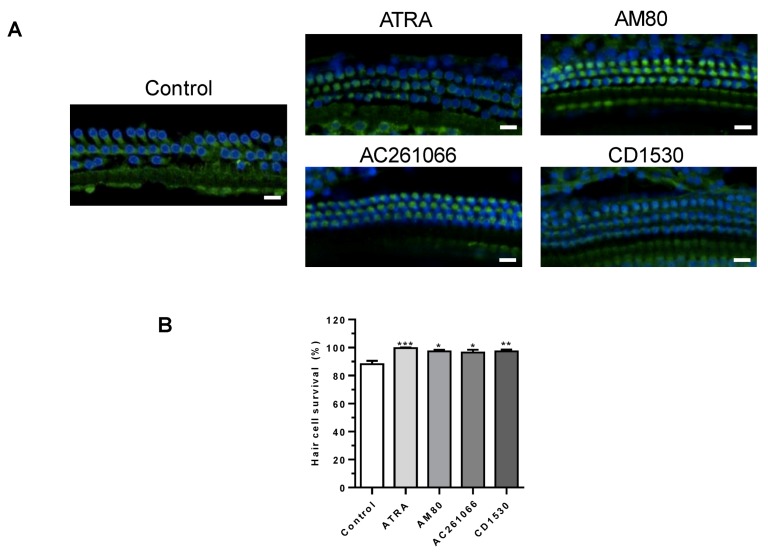
Effect of all-trans retinoic acid (ATRA) on hair cell damage after noise exposure. (**A**) Day 7, whole mount preparation of the cochlea was performed. Hair cells were stained with 4′,6-diamidino-2-phenylindole (DAPI; blue) and Myosin 7a (green), and observed under a confocal microscope (scale bar, 20 µm). Images of middle turn of cochlea, control group showed damaged outer hair cells, whereas the selective retinoic acid receptor (RAR) agonist groups showed preserved outer hair cells. (**B**) Outer hair cell survival rate in each group was compared (*n* = 6–10). Scale bars, 10 µm. Statistical analysis was performed with the reference value of lane 1. *** *p* < 0.001, ** *p* < 0.01, * *p* < 0.05.

Next, hair cell survival was examined at 1 week after noise exposure (Figure 4A). Whole mount preparations of the middle turn of cochlea were performed and examined by confocal microscopy. In the control group, 88.94% of cochlear outer hair cells survived at the middle turn. The RAR agonist-treated groups showed significantly better outer hair cell survivals (Figure 4B). The summary of the data were described in Table 1.

## 4. Discussion

Noise-induced hair cell damage is critical in that it results in temporary or permanent hearing loss. In mammals, damaged hair cells are difficult to regenerate; once a permanent threshold shift has developed, it cannot be repaired. Studies regarding RA treatment for NIHL have shown preservation of hearing in mouse models of permanent and temporary threshold shift. Previous studies have revealed that noise exposure induces the JNK pathway in inner ear cells. JNK is a protein kinase activated by cellular stress; it activates phosphorylation of c-Jun, which mediates the initiation of apoptosis. RA inhibits the induction of JNK, leading to the inhibition of apoptosis in the inner ear hair cells [3,5].

A recent study showed ATRA-induced peroxiredoxin 6 (Prdx6)-associated recovery from NIHL. Increased expression of Prdx6 protects cells from membrane damage associated with peroxidation [5]. Increased Prdx6 protein expression was observed early after noise exposure; it remained elevated for 24 h. The RAR-related orphan receptor α1 is associated with Prdx 6 expression. RAR α functions as a transactivator of Prdx6 gene expression. These findings suggest that selective RAR agonists have specific roles, which differ among each other.

Our results also suggest that temporary noise exposure not only damages hair cells but also decreases auditory neuronal responses, which is referred to as amplitude in wave I in ABR. Decreased amplitude of wave I in ABR after noise exposure is consistent with the previous result that cochlear synaptopathy and the followed cochlear nerve degeneration is associated with temporary NIHL [12,13]. Although hearing threshold in ABR is completely recovered after temporary noise exposure, there is still a decrease in synaptic vesicles in inner hair cells and it may cause hidden hearing loss, which is a potential risk factor for age-related hearing loss in the later. Notably, the present data show that ATRA and selective RAR agonists ameliorate decrease in wave I amplitude in ABR. In this regard, ATRA or selective RAR agonists could be a potential target for the prevention of NIHL after temporary noise exposure.

As mentioned above, RAR distribution in the developing inner ear is quite different. RAR α is predominantly expressed in the developing sensory epithelium, RAR β in inner ear mesenchymal tissues, and RAR γ in the differentiating otic capsule. In an adult mouse, RAR α and RAR γ transcripts are found in the organ of Corti and the spiral ganglion, whereas RAR β transcripts are localized in mesenchyme-derived tissues. However, our result showed no differences between RAR agonist-treated groups. All selective RAR agonists can protect hair cell from noise, especially at high frequencies. There were no statistical differences at Day 7 of the experiment (6 days after noise exposure). We assume that it was a result of small sample size and unexpected loss of mice during the experiment. We performed IP injection with dissolved agonists in DMSO. DMSO is a well-known solvent that can solubilize a wide variety of molecules. We also confirmed that DMSO did not have any effects to cochlea, by comparing with water injected group to rule out placebo effect. There was no difference between water and DMSO groups in ABR threshold, wave I amplitude and hair cell survival rate (Supplementary Figure S1). However, there have been reports regarding the unexpected low-dose toxicity of DMSO [14,15]. During our experiments, some mice were lost in both control and agonist groups for unknown reasons.

Our data did not show any differences between selective RAR agonists; however, other studies have shown that selective RAR agonists can serve as therapeutic options in many diseases. Selective RAR α agonists have therapeutic potential for the treatment of immune diseases, cancer, and Alzheimer’s disease [16]. Selective RAR α agonists have shown inhibition of tumor proliferation, as well as induction of tumor cell apoptosis, in murine models [17]. In T-cell lymphoma, RAR α overexpression augmented chemosensitivity to retinoids and AM80 administration inhibited cell growth, which may indicate a therapeutic target in some peripheral T-cell lymphomas [18]. In addition, the selective RAR α agonist AM80 showed a neuroprotective effect in a mouse model of intracerebral hemorrhage. AM80 decreased activation of macrophages affected by oxidative stress. Treatment with AM80 also decreased the area of nitrotyrosine immunoreactivity [19]. Another study showed that nitrotyrosine immunoreactivity can be induced by noise; notably, increased nitrotyrosine expression in outer hair cells caused apoptosis in a guinea pig model [20]. Therefore, it is highly possible to apply selective RAR α agonist against NIHL.

## 5. Conclusions

RA and selective RAR agonists exert protective effects in NIHL, such that outer hair cells are preserved. Given that most of the environmental noise are in the level of transient threshold shift, we considered that retinoic acid receptor agonists could be utilized to prevent transient shift and relieve the related tinnitus symptom. Regardless of differences among RAR agonists, they all demonstrate protective effects, especially during exposure to high-frequency noise, and may serve as therapeutic agents for NIHL.

## Figures and Tables

**Table 1:** The preventive effects of retinoic acid receptor agonists on noise-induced hearing loss.

Compounds	Hearing Threshold (dB SPL)	Wave I Amplitude (nV)	Hair Cell Survival (%)
Control	92.1 ± 16.8	143.2 ± 365.9	88.2 ± 7.6
ATRA	65.0 ± 22.1 *	471.7 ± 455.7	99.7 ± 1.1 ***
AM80	58.6 ± 9.1 *	637.5 ± 515.7 *	97.4 ± 2.5 *
AC261066	64.6 ± 20.4 *	538.1 ± 452.2	96.5 ± 6.1 *
CD1530	62.9 ± 23.1 *	587.4 ± 561.5 *	97.3 ± 3.4 **

Hearing threshold and wave I amplitude are the results from auditory brainstem response by click stimulation in post-noise 1 day. Statistical analyses were performed in comparison to the control group. * *p* < 0.05; ** *p* < 0.01; *** *p* < 0.001.

## Supplementary Materials

The following are available online at https://www.mdpi.com/1660-4601/16/18/3428/s1. Figure S1: Data from placebo group with water (*n* = 3) was compared with that from placebo group with DMSO (*n* = 3). A. in post-noise day 7, wave I amplitude in auditory brainstem response (ABR) did not differ between two groups. B. ABR threshold by click stimuli after noise exposure were not significantly different between two groups over time.

## Abbreviations

NIHL	Noise induced hearing loss
RA	Retinoic acid
RAR	Retinoic acid receptor
ATRA	All-trans retinoic acid
IP	Intraperitoneal
