# Supplementary material for: Effect of Specific Retinoic Acid Receptor Agonists on Noise-Induced Hearing Loss
Source: Int J Environ Res Public Health. 2019 Sep 16;16(18):3428. doi: 10.3390/ijerph16183428 (PMC6765908; doi:10.3390/ijerph16183428)
Supplement: Supplementary file 1 [file ijerph-16-03428-s001.zip › ijerph-553969-supplementary.pptx]

## Slide 1
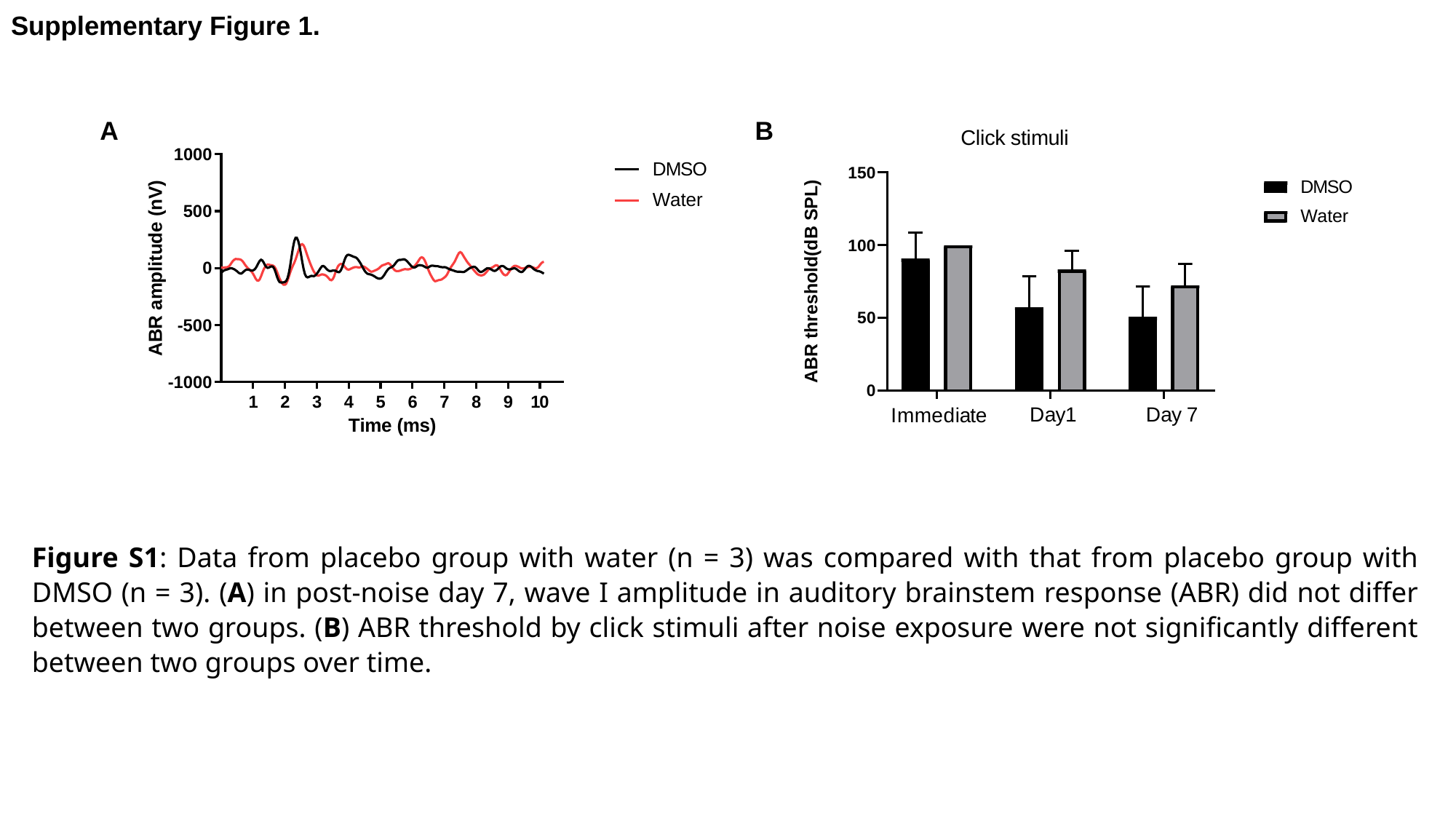

Supplementary Figure 1.
Figure S1: Data from placebo group with water (n = 3) was compared with that from placebo group with DMSO (n = 3). (A) in post-noise day 7, wave I amplitude in auditory brainstem response (ABR) did not differ between two groups. (B) ABR threshold by click stimuli after noise exposure were not significantly different between two groups over time.
